# The role of tetradecane in the identification of host plants by the mirid bugs *Apolygus lucorum* and *Adelphocoris suturalis* and potential application in pest management 

**DOI:** 10.3389/fphys.2022.1061817

**Published:** 2022-12-06

**Authors:** Haichen Yin, Wenjing Li, Min Xu, Dong Xu, Peng Wan

**Affiliations:** Key Laboratory of Integrated Pest Management on Crops in Central China, Ministry of Agriculture and Rural Affairs, Hubei Key Laboratory of Crop Pests and Weeds Control, Institute of Plant Protection and Soil Fertilizer, Hubei Academy of Agricultural Sciences, Wuhan, China

**Keywords:** *Adelphocoris suturalis*, *Apolygus lucorum*, miridae, tetradecane, olfactory responses, pest management

## Abstract

The mirid bugs *Apolygus lucorum* and *Adelphocoris suturalis* are considered serious pests of many crops in China, the host plant recognition of these pests remains unclear. The current study investigated the vital odor cues of two mirid bugs and evaluated the role of olfactory recognition in host recognition. The GC-EAD response of mirid bugs to volatiles of their host plant *Phaseolus vulgaris* was tested. Tetradecane, 2-propyl-1-pentanol, and dodecanal elicited strong EAG responses by mirid bugs and were tested with field experiments. The results indicated tetradecane was significantly more attractive than other attractants, yielding 30.33 ± 2.19 mirid bugs trapped during 7 days. The selected response rates to tetradecane were above 60%, which was most attractive to female *A. lucorum* at 1.5 mg/ml. Among seven tetradecane derivatives, tetradecane and tetradecanoic acid were the most potent attractants to *A. lucorum* and *A. suturalis*. Tetradecane was present in the volatiles of 10 common hosts, and their difference in relative content was significant. The presence of tetradecane seemed relevant to the olfactory response intensity of two mirid bugs towards the different host plants. The artificial supplement of tetradecane increased the attractive effect of host plants. These results suggested that tetradecane plays a vital role in the olfactory selection by two mirid bugs, and it can be made into field baits as a novel ecological strategy to manage these pests with widely reported pesticide resistance. However, results suggested host recognition is not entirely dependent on odor cues. We demonstrated that *A. suturalis* and *A. lucorum* adults have similar olfactory recognition mechanisms to their hosts in long-distance host selection. While, the differences in host plant selection between the two pests should occur in close range due to differences in gustatory or tactile sensory organs of *A. lucorum* and *A. suturalis*.

## Introduction

The mirid bugs *Apolygus lucorum* (Meyer-Dür) and *Adelphocoris suturalis* (Jakovlev) (Hemiptera: Miridae) attack a wide range of cultivated crops, including cash crops such as cotton, jujube, and the kidney bean (Blackmer et al., 2004; Pan et al., 2015a). These bugs were originally minor cotton pests in China. However, since the introduction of transgenic cotton in the late 1990s (Lu et al., 2010; Lu et al., 2012), mirid populations have rapidly increased, becoming significant cotton pests in major cotton-growing regions of the country (Lu and Wu, 2011).

Currently, *A. lucorum* and *A. suturalis* are managed mainly through synthetic insecticides that may negatively impact the environment, non-target insects, and human health. Moreover, these bugs have quickly developed resistance against many insecticides (Snodgrass et al., 2009; Zhen et al., 2016). Some previous studies discovered that *A. lucorum* population in China has moderate resistance to several insecticides (Guo et al., 2010; Zhang et al., 2015). All this limit the use of chemical control. There is, therefore, an urgent need to explore and test alternative management methods for mirid bugs.

Many studies have documented the “chemical conversation” with the surrounding environment by insects using their remarkable olfactory organs (Del Socorro et al., 2010; Gregg et al., 2010; Robert and Iain, 2010; Knight and Light, 2013). Insect pests can select suitable food sources or oviposition sites by odorous cues provided by plants in the form of volatile emissions announcing their physiological status in the natural environment (Shiojiri and Karban, 2008; Weiss et al., 2011; Sun et al., 2012; Cha et al., 2017; Hosseini et al., 2017). Previous study has found some important odorous cues recognized by *A. lucorum* including butyl acrylate, butyl propionate, diethyl phthalate and methyl levulinate in the host plant volatiles (Pan et al., 2015a; Yin et al., 2021). However, compared to the enormous knowledge about lepidopteran and coleopteran pests, very little is known about the responses of mirid bugs, especially *A. suturalis* to plant volatiles (Pan et al., 2015a; Pan et al., 2015b). The two species of mirid bugs have many common host plants we tried to verify whether *A. suturalis* have similar olfactory recognition mechanisms with that of *A. lucorum* or not. Thus in this study, we hope to find the vital odor cues in a wide range of shared host plants of two mirid bugs. In addition, previous studies mainly focused on olfactory recognition we attempt to demonstrate whether host selection is entirely dependent on odor cues by seeking evidence that other sensory organs are involved in host identification. We hope this study will contribute to understanding the host plant recognition mechanism of *A. lucorum* and *A. suturalis* which can be a theoretical basis for developing safe and effective potential field baits for both pests.

The current study tested GC-EAD responses of *A. lucorum* and *A. suturalis* to volatiles of the common bean, *Phaseolus vulgaris* (Fabacea), an important host plant of these pests. The volatiles were identified by gas chromatography and mass spectroscopy (GC-MS). In field tests and laboratory trials, lure trapping and two-way competition experiments were used to examine the relative attractiveness of substances that produced EAD responses. Finally, the attractive effects of ten other host plants on the insects were also evaluated by ‘strictly olfactory’ assays and normal assays. Then we analysed the relationship between behavioral responses and the composition of volatiles. The key objective of this study was to understand the mechanisms of host plant identification used by two mirid bugs towards developing specific trapping protocols for controlling field infestations.

## Materials and methods

### Reagents and instruments

Synthetic analogues of the volatiles identified from *P. vulgaris* were purchased from Shanghai Macklin Biochemical Co., Ltd., and Aladdin Industrial Corporation, China. A three-arm olfactometer (Brand: YMM3-150), a GC-EAG measurement system, and a QP 2010 SE GC-MS system were purchased from Shanghai Yuming Instrument Co., Ltd., Syntech Limited, and Shimadzu Corporation, respectively. The adsorption column consisted of a glass tube (15 cm long and 0.7 cm in diameter) with an airflow outlet and inlet equipped with 200 mg SuperQ adsorbent, purchased from Altec, United States.

### The collection of volatiles of *P. vulgaris* pods

About 15 *P. vulgaris* pods were placed inside a glass jar (35 × 20 cm – height and diameter) provided with an airflow inlet and outlet at the top. An adsorption column was attached to the outlet exit, while an activated carbon filter (air flow rate: 600 L/min) was attached prior to the inlet. After collecting air volatiles for 8 h, the adsorption column was removed to be eluted with GC-MS grade hexane.

### The GC-EAD response of *A.lucorum and A.suturalis* to volatiles of *P. vulgaris* pods

Two 4.5 cm long glass capillaries were filled with 0.9% sodium chloride saline and inserted with two 4.5 cm long clean silver wires as electrodes. One glass tube was used as the measuring pole and the other one served as the reference pole.

Antennae of adult bugs were excised at the base using a scalpel under a dissection microscope and chipped at their tip. One antenna was attached to the reference pole by the surface tension of the saline solution and connected to the measuring pole under a stereo microscope. When the baseline readings of the EAD remained stable and the GC oven was ready. 4 μl of plant volatile solution was injected into the GC port. Obtained data were recorded and analyzed with GC-EAD 2014 V1.2.5 software.

### Identification of volatiles of *P. vulgaris* pods

The procedure for collecting volatiles was as previously stated. Gas chromatograph settings included an Agilent DB-wax chromatographic column (30 m. 0.25 mm; 0.25 µm, Agilent Technologies). The injector temperature was set to 225°C, carrier gas was helium, and injections were made on splitless mode. The oven temperature was programmed to three steps: 1) 40°C hold for 5 min; 2) increase at the rate of 5°C/min to 110°C, hold for 1 min; 3) increased at the rate of 10°C/min to 240°C and hold for 10 min. The mass spectrometer ion source temperature was set to 300°C, and the scanning speed used at 2500, with a 0.3 s interval. Obtained mass spectra were compared with external synthetic standards using Labsolution software to confirm the chemical identity of the main volatiles recovered from *P. vulgaris* pods.

### Attractive effects of volatiles from *P. vulgaris* pods under field conditions

Volatiles eliciting significantly stronger EAG responses were selected for attractiveness field tests using synthetic analogues. The field tests were conducted from 28 August to 3 September 2020 in a cotton field measuring about 4,000 m^2^ in Ezhou, Hubei, China. Respective synthetic compounds were diluted to 150 mg/ml with ddH_2_O, and 1 ml solution was added to individual micro centrifuge tubes sealed with plastic film, minutely punctured to prevent quick evaporation. The tubes were taped to the middle of a yellow board (RAL 1016 Sulfur yellow according to the international standard colour metric scale). Glue was spread on the outer face of the yellow board to capture the bugs. Each compound was tested three times using different yellow boards. Three boards were presented parallel as controls containing no attractants, using ddH_2_O as a control. Treatment and control groups were randomly distributed, distanced from each other by at least 20 m, where each board was held suspended at about 20 cm above a cotton plant. After 7 days, the numbers and species of mirid bugs captured on the yellow boards were counted.

### Attractive effects of volatiles from *P. vulgaris* pods in laboratory tests

**FIGURE 1:**
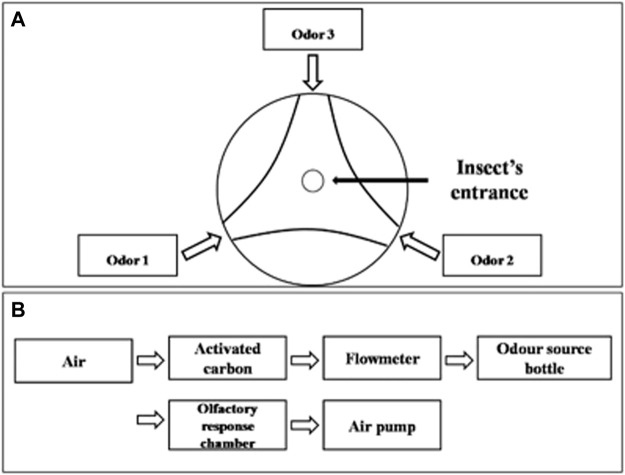
Schematic diagram of YMM 3–150 olfactometer. **(A)** Top view of olfactory reaction chamber of olfactometer, **(B)** connections diagram of olfactometer.

Volatiles presenting a positive attractiveness response in field experiments were further tested by a two-way competition experimental design in laboratory conditions. Prior to the tests, male and female adults of *A. suturalis* and *A. lucorum* were starved for 4 h and submitted to olfactory behavior assays using a three-arm olfactometer (Figure 1). The arms were ventilated for 5 min before the experiment, with the airflow rate set to 2 L/min. The synthetic volatiles were diluted to 1.5 mg/ml and 15 mg/ml in ddH_2_O and added 30 μl anhydrous ethanol to assist in complete solubilization. One of the olfactometer arms was blocked with absorbent cotton, and the other two were connected to glass bottles containing either 50 μl volatile solution or ddH_2_O with 30 μl anhydrous ethanol as a control group. Ten adult bugs were placed in the response chamber from an opening at the top. The test was repeated nine times for each species, totaling 90 male and female adults assayed for each species. All behavioral response assays were done in the dark, being recorded for 30 min once insects were introduced. Any insects moving beyond 50% of an arm’s length were considered to have shown a positive attractiveness response to the tested volatile. In setting up each replicate, the olfactometer parts were washed with ethanol, and the direction of odor sources were randomized.

The selected response rate and selection coefficient were calculated as follows (Zhou et al., 2009):

$$Selected\;response\;rate\;=\frac{No.\;adults\;selected\;treatment\;}{\left(No.adults\;selected\;treatment+No.adults\;selected\;control\;\right)}\times 100$$

$$Selection\;coeficiant\;=\frac{\left(Number\;of\;adults\;selected\;treatment-Number\;of\;adults\;selected\;control\right)}{\left(Number\;adults\;selected\;treatment+Number\;of\;adults\;selected\;control\right)}$$

### Attractiveness to tetradecane analogues by *A. lucorum* and *A. suturalis* adults

To test the attractiveness effects of tetradecane analogues, seven analogues, including aldehydes, aldehydes, alcohols and acids, were employed for two-way competition experiments of female adults as described above. The analogues were 7-tetradecene, 1-tetradecene, tetradecanal, 2-tetradecanol, 1-tetradecanol, 2-tetradecenoic acid and tetradecanoic acid.

### Tetradecane amounts in the bouquet of other selected host plants, and attractiveness to bugs

Fruits of *Vitis vinifera*, *Zea mays*, *Pyrus bretschneideri*, *Malus pumila* and seedlings of *Pisum sativum* (100 g each) were placed in individual glass jars to collect volatiles, as previously described. Five different plant parts of cotton, including buds, bolls, seedling leaves, bolls leaves, and bud leaves, were obtained from Xinzhou in 2019 and stored in afrigerator at −80°C as ground powders. Fifteen grams of each different plant part sample were likewise used to collect volatiles.

Volatile compounds were identified by GCMS, but using a Rtx-5 MS chromatographic column (30 m × 0.25 mm x 0.25 µm), injection port temperature was 250°C, helium as the carrier gas, injections done in splitless mode. The oven temperature program was set to three steps: 1) 40°C for 1 min; 2) increasing at the rate of 4°C/min to 130°C hold for 5 min; 3) increasing at the rate of 10°C/min to 250°C, final hold for 5 min. The mass spectrometer ion source temperature was set to 250°C; the scanning speed was 2500 with a 0.3 s interval. Runs were repeated three times, and the relative content of tetradecane in each sample was based on obtained peak areas in respective chromatograms.

Further 15–20 g of *Vitis vinifera*, *Zea mays* fruits, and 5-7 cotton leaves were employed to test for attractiveness to mirid bugs, but these tests were designed as ‘strictly olfactory’ assays and normal assays using female adults. In the case of the ‘strictly olfactory’ assays, three different plants were presented as host options inside separate glass bottles connected to three arms of the olfactory response chamber and the odor of host plants was blown into the response chamber from glass bottles by filtered air so the mirid bugs could smell them but could not reach the host plant through the hose that connected the response chamber and the glass bottles. Hence, they were unable to access them visually or physically. Evaluation methods were as described above.

In the case of normal assays, the three samples of different plant parts were offered as host options directly inside the different arms so that mirid bugs could evaluate hosts by smell, touch and gustation cues because in this case there is no barrier between the host plants and pests. Twenty adult *A. suturalis* and A. lucorum were placed into the olfactory response chamber from an opening at the top centre in each replicate and left overnight between 17:00–8:00. Lights were switched off to maintain dark conditions. Each assay was repeated three times, where the positions of host options were changed and randomized. Response rates were calculated as described previously.

### Olfactory responses of *A. lucorum* and *A. suturalis* to plants supplemented with tetradecane

Plants presenting the highest and lowest tetradecane contents were selected for this experiment. The plant with the lowest tetradecane content was supplemented with added synthetic tetradecane to quantify any relative attractiveness changes using the three-arm olfactometer described above. In this experiment, one treatment group and two control groups were set up and 15–20 g of the host plants were used in each group. The treatment group contained host plants with the lowest tetradecane content, and the plants were artificially supplemented with 50 μl of tetradecane solution. Control group 1 included host plants containing the highest tetradecane contents, and Control group 2 included host plants containing the lowest tetradecane content but no artificial tetradecane supplement. Three different concentrations of tetradecane supplementation were tested: undiluted solution, 15 mg/ml and 1.5 mg/ml. Treatment and two control groups were thus allocated to the three glass bottles connected to the olfactometer to be assayed as described above in ‘strictly olfactory’ experiments.

### Data analysis

The Least Significant Difference (LSD) test was employed to check for differences in the numbers of mirid bugs trapped by boards presented in the field, in the content of tetradecane in each examined plant bouquet, and in the relative attractiveness of different plants to hosts. Olfactory responses to volatiles and analogues were analyzed using the chi-square test. All analyses were conducted using SPSS software, while data of GC-EAD were analyzed with GC-EAD 2014 V1.2.5 software.

## Results

### The GC-EAD responses of *A.lucorum and A.suturalis* to volatiles from *P. vulgaris* pods

**FIGURE 2:**
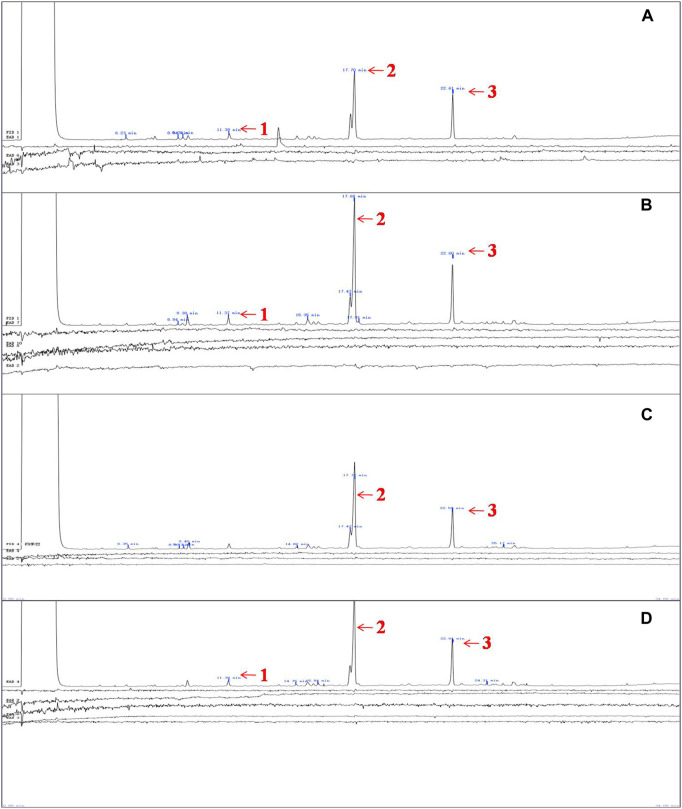
Measured EAG responses of A. lucorum and *A. suturalis* to volatiles from *Phaseolus vulgaris*. **(A)** The EAG responses of female *A. lucorum* to volatiles from *P. vulgaris*, **(B)** The EAG responses of female *A. suturalis* to volatiles from *P. vulgaris*, **(C)** The EAG responses of male *A. lucorum* to volatiles from *P. vulgaris*, **(D)** The EAG responses of male *A. suturalis* to volatiles from *P. vulgaris* 1, 2,3 indicated FID peaks of three volatiles at 11.39 min, 17.70 min, and 22.61 min, approximately, which induced EAG responses of **(A)** lucorum and *A. suturalis* adults.

The results of GC-EAD indicated three compounds from *P. vulgaris* pods inducing antennal responses in males of *A. suturalis*, and females of *A. suturalis* and *A. lucorum.* Their respective retention times were 11.39 min, 17.70 min and 22.61 min (Figure 2), corresponding to 2-propyl-1-pentanol, tetradecane and dodecanal (Table 1), respectively. Interestingly, 2-propyl-1-pentanol did not induce any EAG response in *A. lucorum* males.

**TABLE 1:** Volatiles from common beans *Phaseolus vulgaris* pods, identified by gas-chromatography coupled with mass spectrometry.

PK	Retention time (min)	Area percentage (%)	Library/ID	CAS	Quality
1	3.1843	9.7341	12-Crown-4	000294-93-9	64
2	4.0953	5.3682	Trichloromethane	000067-66-3	76
3	7.1804	4.3985	15-Crown-5	033100-27-5	59
4	7.2703	0.588	1,4,7,10,13,16-Hexaoxacyclooctadecane	017455-13-9	43
5	7.4578	1.1444	o-Xylene	000095-47-6	42
6	9.1109	0.6007	3,6,9,12-Tetraoxatetradecan-1-ol	005650-20-4	43
7	9.3508	2.0295	D-Limonene	005989-27-5	92
8	10.0931	0.4811	Octaethylene glycol monododecyl ether	003055-98-9	58
9	10.6366	0.5729	2-Butanol, 3-(1-methylbutoxy)-	074810-43-8	38
10	11.6675	3.0992	1-pentanol,2-propyl-	058175-57-8	94
11	14.2541	3.0293	Benzene, 1,2,3,4-tetramethyl-	000488-23-3	55
12	15.2025	0.972	1-Undecanamine	007307-55-3	43
13	15.956	1.0431	Oxirane, 2-butyl-3-methyl-, cis-	056052-93-8	43
14	17.4067	0.5206	Benzene, 1-methyl-2-(1-methylethyl)-	000527-84-4	90
15	17.8903	26.3347	Tetradecane	000629-59-4	93
16	18.1302	7.6734	Bicyclo [2.2.1]heptan-2-one, 1,7,7-trimethyl-, (1R)-	000464-49-3	98
17	20.3868	0.741	3-Ethoxy-1,1,1,5,5,5-hexamethyl-3-(trimethylsiloxy)trisiloxane	018030-67-6	32
18	21.1291	0.8359	Methoxyacetic acid, 4-tridecyl ester	1000282-04-7	49
19	23.0259	24.2746	Dodecanal	000112-54-9	89
20	23.6557	0.4375	Pentonic acid, 5-deoxy-2,3-bis-O-(trimethylsilyl)-, .gamma.-lactone	074742-33-9	32
21	24.7727	1.024	Naphthalene, 1-methyl-	000090-12-0	90
22	25.1813	0.4541	Propanoic acid, 2-methyl-, 2-ethyl-3-hydroxyhexyl ester	074367-31-0	35
23	25.6087	1.0167	Bicyclo [2.2.1]heptane-2-methanol	005240-72-2	38
24	25.7511	0.2625	Mercaptoacetic acid, bis(trimethylsilyl)-	006398-62-5	40
25	26.6471	0.4437	Naphthalene, 1,3-dimethyl-	000575-41-7	68
26	29.8559	0.4162	Oxirane, 2-methyl-3-propyl-, cis-	006124-90-9	55
27	30.7293	0.5118	Methoxyacetic acid, 3-pentadecyl ester	1000282-05-2	53
28	31.5653	0.2927	Octaethylene glycol monododecyl ether	003055-98-9	53

### Attractive effects of volatiles from *P. vulgaris* pods under field conditions

**FIGURE 3:**
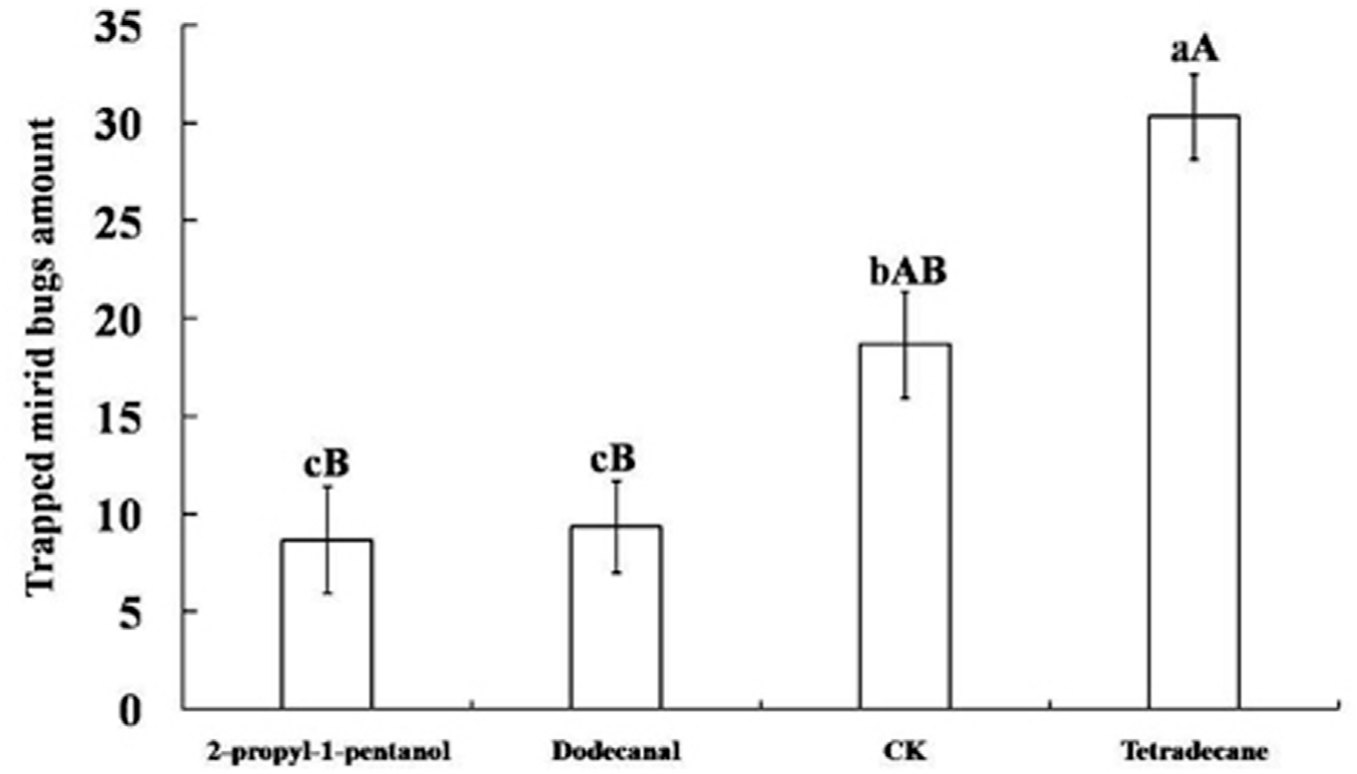
Mirid bugs trapped by yellow boards with different attractant. Different uppercase and lowercase letters indicated extremely significant (*p* < 0.01) and significant (*p* < 0.05) differences of mirid bugs amount that trapped by yellow boards with three different attractants and control group (*Least significant difference test*).

Tetradecane, 2-propyl-1-pentanol, and dodecanal were obtained as synthetic compounds for field tests as baits on yellow board traps. As shown in Figure 3, tetradecane yielded high attractiveness: within 7 days, the average amount of captured mirid bugs was 30.33 ± 2.19, significantly more than with other attractants (*p* < 0.01) and controls (*p* < 0.05). In fact, the number of captured mirid bugs trapped by 2-propyl-1-pentanol and dodecanal was significantly lower than in controls (*p* < 0.05).

### Confirmation of the attractiveness of compounds in lab experiments

In field experiments, tetradecane appeared as the most attractive volatile to bugs; nevertheless, many uncontrollable factors may have influenced these findings, necessitating confirmation in a laboratory setting. In two-choice olfactory tests, volatile concentrations of 1.5 mg/ml and 15 mg/ml were used. Results showed that tetradecane significantly increased female mirid bugs’ attractiveness, with selected response rates above 60%. Tetradecane was most effective in attracting females of both bug species at a concentration of 1.5 mg/ml. While tetradecane was less attractive to males, it showed no significant olfactory response at the concentration of 1.5 mg/ml (Table 2).

**TABLE 2:** The olfactory responses of two pest mirid bugs to synthetic tetradecane, measured in laboratory tests.

Gender	Species	Concentration (mg/mL)	Treatment	Control	NR	*p*-Value	χ^2^	SRR (%)	SC
Female	*Apolygus lucorum*	15	38	24	28	0.012**	6.32	61.29	0.23
		1.5	45	20	25	<0.001**	19.23	69.23	0.38
	*Adelphocoris suturalis*	15	38	23	29	0.007**	7.38	62.30	0.25
		1.5	40	24	26	0.005**	8.00	62.50	0.25
Male	*Apolygus lucorum*	15	45	20	25	<0.001**	19.23	69.23	0.38
		1.5	31	30	29	0.8563	0.03	50.82	0.02
	*Adelphocoris suturalis*<	15	41	22	27	<0.001**	29.30	65.08	0.30
		1.5	31	29	30	0.715	0.13	51.67	0.03

**Represents a highly significant difference between the number of mirid bugs attracted to the treatment group orcontrol group (*p* < 0.01) (*Chi-square test*). NR represents mirid bugs presenting no response, SRR represents selected response rate, and SC represents selected coefficient.

### Attractiveness of tetradecane analogues to *A. lucorum* and *A. suturalis* adults

Seven synthetic derivatives from various chemical groups, including alcohols, aldehydes, acids and olefins, were chosen for the two-way competition experiment to assess the relative attractiveness of tetradecane analogues to *A. lucorum* and *A. suturalis*. As tetradecane proved comparatively less attractive to males, we tested the olfactory response of females only. Overall, tetradecane derivatives proved most attractive to *A. lucorum* than to *A. suturalis*, especially at the lowest concentrations. Four analogues showed stronger attractiveness effects on *A. lucorum* (*p* < 0.001) compared to controls at the concentration of 1.5 mg/ml. Among them, tetradecanal was the most attractive to *A. lucorum*, eliciting a higher selected response rate than tetradecane. Only tetradecanoic acid yielded a higher attraction (*p* < 0.001) of *A. suturalis* at the concentration of 15 mg/ml (Table 3).

**TABLE 3:** The olfactory responses of two pest mirid bugs to synthetictetradecane derivatives, measured in laboratory tests.

Species	Concentration (mg/mL)	Odor source	Treatment	Control	NR	*p*-Value	χ^2^	SRR (%)	SC
*Apolygus lucorum*	15	7-tetradecene	43	22	25	<0.001**	13.57	66.67	0.33
		1-tetradecene	40	25	25	0.001**	10.45	61.72	0.23
		Tetradecanal	29	34	27	0.37	0.79	45.83	−0.08
		2-tetradecanol	33	27	30	0.27	1.2	55.00	0.10
		1-tetradecanol	31	34	25	0.6	0.28	46.94	−0.06
		2-tetradecenoic acid	27	37	26	0.07	3.13	42.42	−0.15
		Tetradecanoic acid	35	25	30	0.07	3.33	58.97	0.18
	1.5	7-tetradecene	44	20	26	<0.001**	18	68.18	0.36
		1-tetradecene	32	34	24	0.73	0.12	48.57	−0.03
		Tetradecanal	48	17	25	<0.001**	29.57	73.33	0.47
		2-tetradecanol	42	25	23	0.003**	8.63	63.16	0.26
		1-tetradecanol	32	31	27	0.86	0.03	51.35	0.03
		2-tetradecenoic acid	33	24	33	0.09	2.84	57.89	0.16
		Tetradecanoic acid	43	23	24	<0.001**	12.12	64.86	0.30
*Adelphocoris suturalis*	15	7-tetradecene	30	31	29	0.86	0.03	48.48	-0.03
		1-tetradecene	31	28	31	0.58	0.3	52.63	0.05
		Tetradecanal	33	29	28	0.47	0.52	53.85	0.08
		2-tetradecanol	32	28	30	0.47	0.53	52.78	0.06
		1-tetradecanol	29	35	26	0.29	1.125	44.83	−0.10
		2-tetradecenoic acid	35	28	27	0.21	1.56	54.84	0.10
		Tetradecanoic acid	49	14	27	<0.001**	38.89	77.78	0.56
	1.5	7-tetradecene	33	28	29	0.37	0.82	54.55	0.09
		1-tetradecene	32	28	30	0.47	0.53	53.19	0.06
		Tetradecanal	32	30	28	0.72	0.13	51.85	0.04
		2-tetradecanol	32	28	30	0.47	0.53	46.51	−0.07
		1-tetradecanol	32	31	27	0.86	0.03	51.16	0.02
		2-tetradecenoic acid	33	29	28	0.47	0.52	53.19	0.06
		Tetradecanoic acid	31	29	30	0.72	0.13	51.61	0.03

**Represents a highly significant difference between the number of mirid bugs that were attracted to the treatment group or control group (*p* < 0.01) (*Chi-square test*). NR represents mirid bugs of no response, SRR represents the selected response rate, and SC represents the selected coefficient.

### Tetradecane content in different host plants and relative attractiveness

**FIGURE 4:**
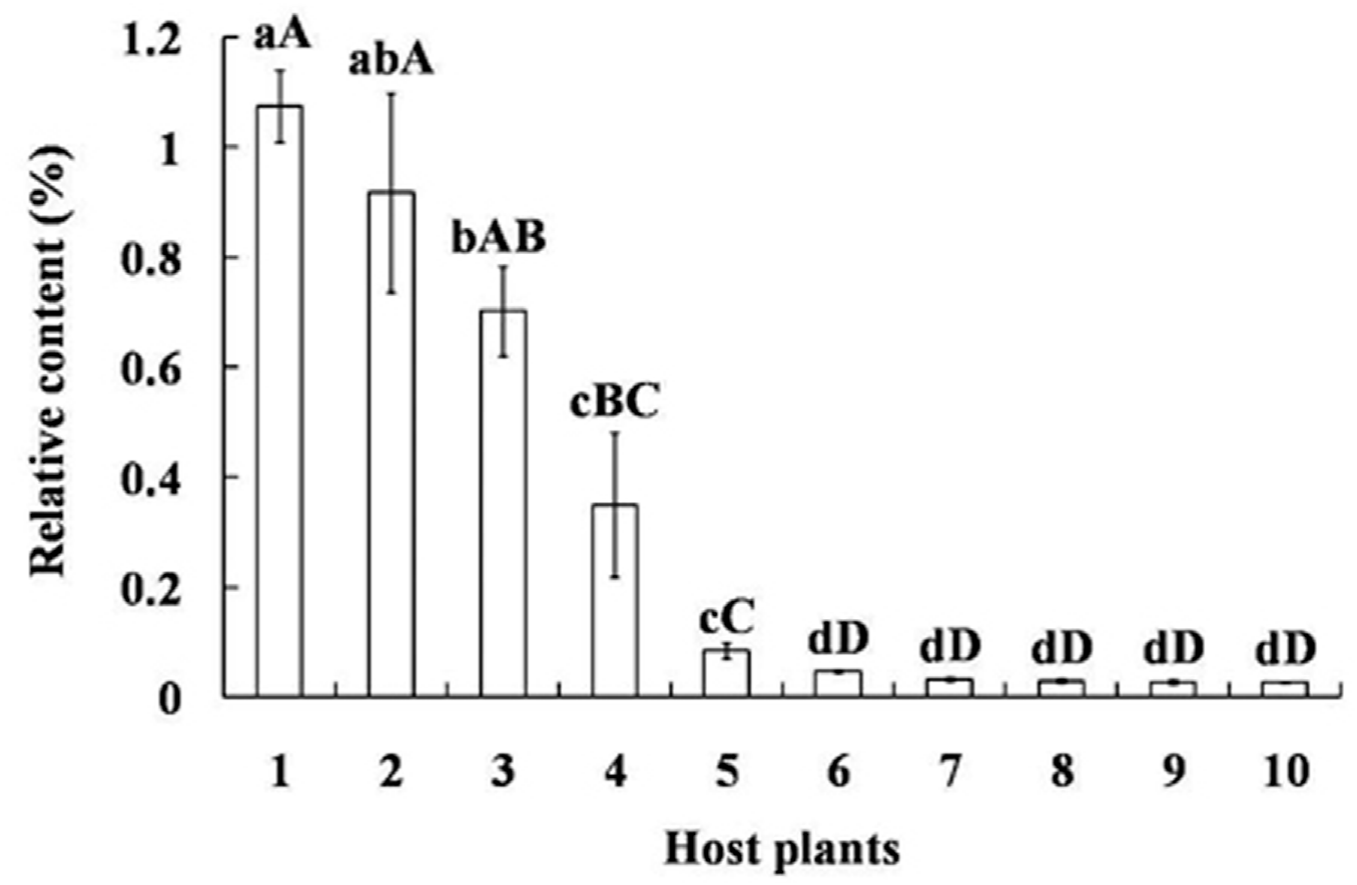
Relative content of tetradecane among volatiles of different plants exploited as hosts by mirid bugs. Different uppercase and lowercase letters indicated highly significant (*p* < 0.01) and significant (*p* < 0.05) differences in the relative contents of tetradecane among ten host plants (*Least significant difference test*). Numbers on the horizontal axis represent: 1. Fruits of *Vitis vinifera*, 2. Seedling of *Pisum sativum*, 3. Fruits of *Zea mays*, 4. Fruits of *Pyrus bretschneideri*, 5. Fruits of *Malus pumila*, 6. Buds of *Gossypium herbaceum*, 7. Bolls of *G. herbaceum*, 8. Leaves at seedling stage of *G. herbaceum*, 9. Leaves on the bolls of *G. herbaceum*, 10. Leaves on the buds of *G. herbaceum*.

GC-MS analysis showed tetradecane is present as a volatile in the other ten plant species commonly exploited by mirid bugs and their relative amounts are significantly different (*p* < 0.05). The concentration of tetradecane was the highest in *V. vinifera* grapes, reaching 1.07 ± 0.06%, followed by *P. sativum* seedlings and *Z. mays* fruits (*p* < 0.05). Other species had statistically similar, lower amounts, including five different plant parts of *G. herbaceum* (*p* < 0.01) (Figure 4).

Therefore, fruits of *V. vinifera* and *Z. mays* and seedling leaves of *G. herbaceum* were selected to test for attractiveness to *A. suturalis* and A. lucorum adults as representative of significantly different tetradecane concentrations. Since tetradecane proved most attractive to females, experiments were designed with females only. Selected response rates of mirid bugs to three host plants in a “strictly olfactory” experiment were correlated with the tetradecane concentration of their volatiles. The selected response rate of A. lucorum to *V. vinifera* was the highest and significantly higher than that of *G. herbaceum* (*p* < 0.01), reaching 44.44 ± 0.80%. While for *A. suturalis*, there were no significant differences between the selected response rates for *V. vinifera* and *Z. mays*, reaching 37.98 ± 2.00% and 38.04 ± 0.54%, respectively. The selected response rate of *G. herbaceum* was significantly lower than that of the other two host plants (*p* < 0.01), reaching 23.97 ± 1.60%.

**FIGURE 5:**
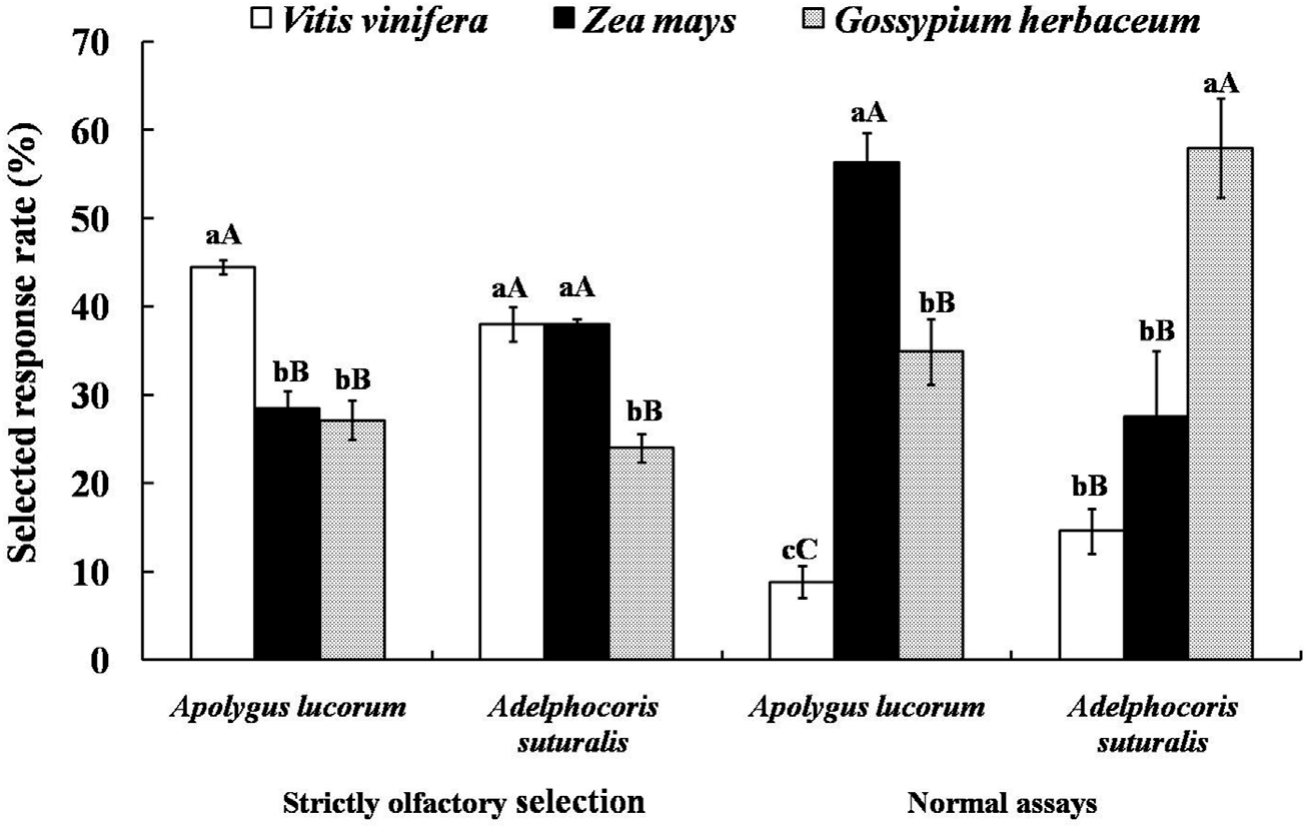
Attractive effects of different host plants on *A. suturalis* and A. lucorum. Different uppercase and lowercase letters showed extremely significant (*p* < 0.01) and significant (*p* < 0.05) differences of the selected response rate of mirid bugs to different hosts in two modes of behaviour tests respectively (*Least Significant Difference test*).

Interestingly, the selected response rates in normal assays (i.e., where bugs can select hosts using multiplecues) were irrelevant with relative contents of tetradecane in the volatiles from three host plants: A. lucorum preferred *Zea mays* fruits, while *A. suturalis* preferred *G. herbaceum* leaves, with respective selected response rates of 56.33 ± 3.30% and 57.92 ± 5.61% (Figure 5).

### Olfactory responses of *A. lucorum* and *A. suturalis* to host plants supplemented with tetradecane

**FIGURE 6:**
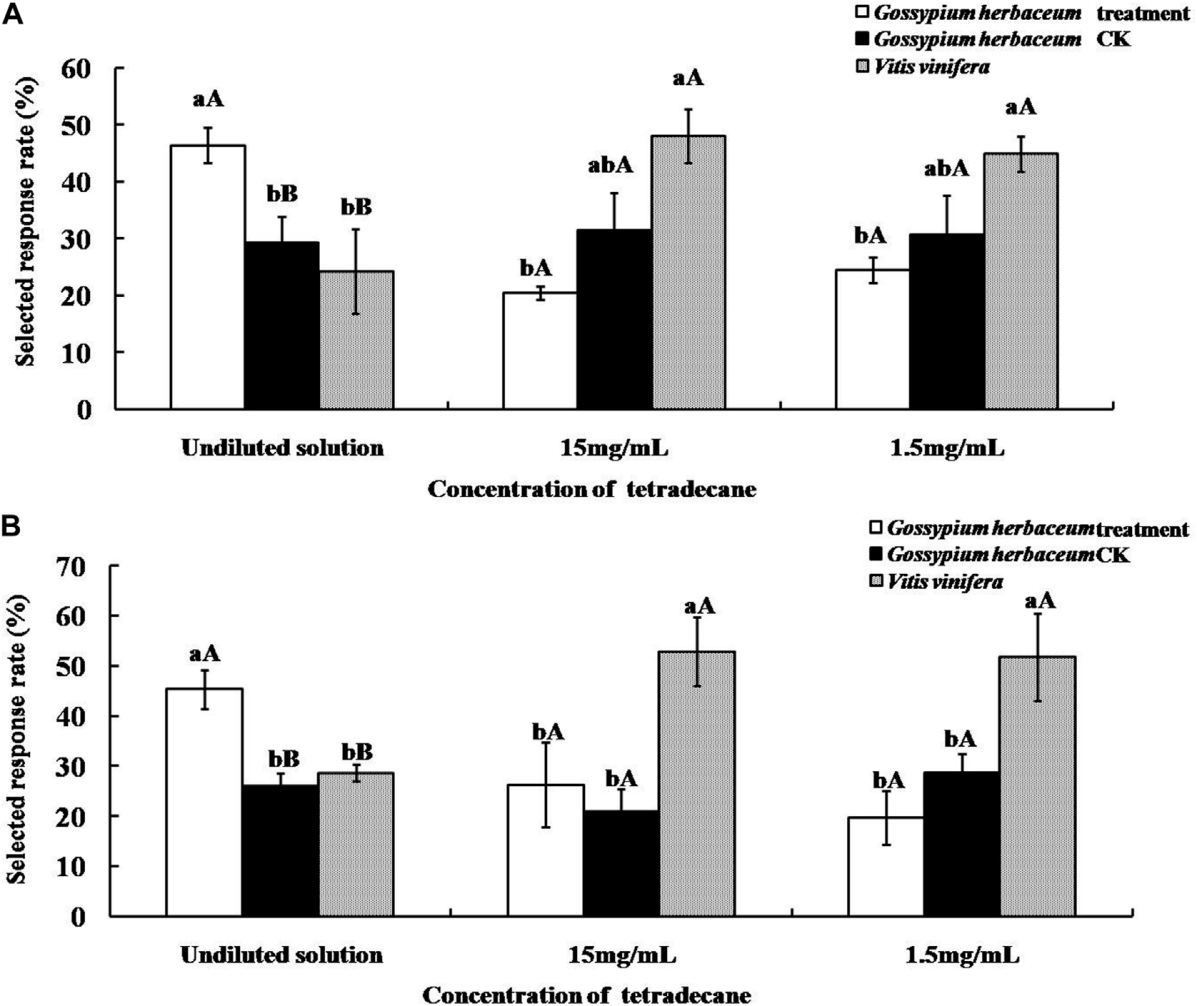
Selected response rates of *Apolygus lucorum* **(A)** and *Adelphocoris suturalis* **(B)** to host plants supplemented with tetradecane. Different uppercase and lowercase letters showed highly significant (*p* < 0.01) and significant (*p* < 0.05) differences in the selected responserate of mirid bugs to different hosts treated by tetradecane of different concentration respectively (*Least Significant Difference test*).

As shown in Figure 6, supplementation with the undiluted solution of tetradecane significantly increased the olfactory selected response rates of both *A. lucorum* and *A. suturalis* to *G. herbaceum* leaves (46.41 ± 3.15% and 45.31 ± 3.86%, respectively). This response was significantly higher (*p* < 0.01) than the pertinent control group and more elevated than *V. vinifera* fruits. On the other hand, when tetradecane was added at concentrations of either 15 mg/ml or 1.5 mg/ml to *G. herbaceum* leaves, response rates did not change significantly (*p* > 0.05).

## Discussion

The current study used laboratory and field experiments to test the volatiles of host plants’ attractiveness in two mirid bugs that might be useful in an attract-and-kill system for these species. Results showed that three volatiles from *P. vulgaris* pods successfully induced EAG responses of *A. suturalis* and *A. lucorum* adults. We further confirmed the attractive effect of tetradecane, which was a common volatile among ten host plants exploited by mirid bugs, by field trapping studies and olfactory lab testing. Additionally, we found that artificially supplementing with tetradecane improved the response rate of host plants with low tetradecane contents. However, it was valid only for olfactory selection.

Previous studies on attractants of mirid bugs are relatively few and mainly focused on *A. lucorum*. Some compounds, including *cis*-formic acid-3-hexene ester, m-xylene, and 3-ethyl benzene have been demonstrated and potentially used to control *A. lucorum* (Sun et al., 2014; Pan et al., 2015a; Pan et al., 2015b). However, the host recognition mechanisms of mirid bugs, especially *A. suturalis* have remained unclear.

The current study showed that tetradecane is a common volatile of the host plants that mirid bugs exploit. Tetradecane is a straight-chain alkane that has a role as a plant metabolite and a volatile component. It also functions as a potent defence induction signal that prepares neighbouring plants for incoming attacks from insect pests (Pan et al., 2022). Some herbivore-infested plants produce tetradecane, which acts as kairomone to attract the pest’s natural enemies (Li et al., 2022). It has been reported as an attractive plant volatile for several insect pests (Mu et al., 2012; Mitra et al., 2020; Chen et al., 2022; Guo et al., 2022). There is, however, scant evidence from earlier investigations that it is an attractant for mirid bugs. When *A. suturalis* and A. lucorum were allowed to select hosts solely by olfaction in ‘strictly olfactory’ assays, attractiveness seems closely related to their relative amounts of tetradecane in volatiles, supporting the hypothesis that tetradecane is an important olfactory clue for mirid bugs. Increased selected response rates further confirmed this after artificial supplementation with tetradecane in host plants presenting low tetradecane concentrations. A possibility for creating attractants centred on a similar chemical structure was also indicated by some attraction displayed by other tetradecane derivatives. We also discovered that male and female mirid beetles react differently to tetradecane. This agrees with previous studies showing that host plant volatiles were more attractive to female adults, while sex pheromones were often more strongly responded by males than by females in locating their mating partners (Groot et al., 2005; Nurkomar et al., 2017).

However, host recognition by insects is a complex process involving different sensory organs at different spatial scales (Bruce et al., 2005; Henze et al., 2018). In the current study, we found that the selected response rates of mirid bugs to three host plants were unrelated to tetradecane contents when mirid bugs were able to contact or taste the hosts contrary to the results in ‘strictly olfactory’ assays. It proved that olfactory recognition is only one aspect of host recognition by mirid bugs, and other sensory organs may also be crucial in host recognition. We considered that different sensory organs are used in field orlaboratory tests except for olfactory organs. In a field experiment, physical signals recognized by visual organs can synergistically enhance host plants’ attractiveness due to the existence of natural light (Kerr et al., 2017). A previous study showed that *A. lucorum* was more attracted to cotton plants containing green lights, and the selected response was significantly higher than that of olfactory or visual cues alone (Pan et al., 2015c). Thus, we believe yellow boards may have contributed synergistically to the attractive effect of compounds in our field experiments.

However, visual cues likely had minimum relevance in our laboratory tests because the experiments were conducted in the dark. In the quite different scenario from the field experiments, gustatory and tactile cues may play a critical role in host recognition which has been demonstrated for several species of herbivorous arthropods (Zhang et al., 2010; Ambrosio and Brooks, 2011). We hypothesize that mirid bugs locate hosts by olfactory cues and settle their selection depending on the contact or taste of a prospective host at close range in the laboratory tests. In this study, we demonstrated that *A. suturalis* and *A. lucorum* adults used the same odor cue, tetradecane, in long-distance host selection, indicating that the two pests have similar olfactory recognition mechanisms to their hosts. However, the differences in host selection between the two pests to *Zea mays* fruits and *G. herbaceum* leaves should occur in close range after contacting or tasting them, which may indicate differences in gustatory and tactile sensory organs of two mirid bugs. Future studies could focus on establishing the role of the various sensory organs, especially gustation and tactile receptors, in recognizing plant hosts towards developing even more effective field traps.

To date, insect attractants proven effective in pest management are mainly sex pheromones (Rizvi et al., 2021). While being more attractive to female mirid bugs, we think tetradecane might also supplement the effects of some sex pheromones in field applications. The baits yielded significant attractiveness when applied with yellow boards in the field. To further enhance the impact as a attract-and-kill products, it is an excellent strategy to combine this attractant with sex pheromones (Kendra et al., 2017; Baroffio et al., 2018; Tasin et al., 2018). Previous studies showed that sex pheromones enhance the attractiveness of host plant volatiles to males. The host plants’ volatiles can increase males’ EAG response to sex pheromones and promote mating behaviour (Binyameen et al., 2013; Ian et al., 2017). However, further work would be needed to optimize the blends of pheromones and plant volatiles, their stability in the field and their effectiveness. We hope the current study contributes to developing more efficient and safer pest control strategies against mirid bugs in the future.

## Data Availability

The original contributions presented in the study are included in the article/Supplementary Material, further inquiries can be directed to the corresponding author.
